# The Omics Hunt for Novel Molecular Markers of Resistance to *Phytophthora infestans*

**DOI:** 10.3390/plants11010061

**Published:** 2021-12-25

**Authors:** Hana Dufková, Miroslav Berka, Marie Greplová, Šarlota Shejbalová, Romana Hampejsová, Markéta Luklová, Jaroslava Domkářová, Jan Novák, Viktor Kopačka, Břetislav Brzobohatý, Martin Černý

**Affiliations:** 1 Department of Molecular Biology and Radiobiology, Faculty of AgriSciences, Mendel University in Brno, 61300 Brno, Czech Republic; dufkova.ha1@gmail.com (H.D.); miroslavberka94@gmail.com (M.B.); sarlotashejbalova@gmail.com (Š.S.); luklovam@gmail.com (M.L.); novakhonza@atlas.cz (J.N.); brzoboha@ibp.cz (B.B.); 2 Potato Research Institute, Ltd., 58001 Havlíčkův Brod, Czech Republic; greplova@vubhb.cz (M.G.); hampejsova@vubhb.cz (R.H.); domkarova@vubhb.cz (J.D.); 3 VESA Velhartice a.s., 34142 Kolinec, Czech Republic; kopacka@vesa-velhartice.cz

**Keywords:** *Phytophthora infestans*, late blight, resistance, proteome, metabolome, lipidome, resistance markers, Oomycetes, *Oomycota*

## Abstract

Wild *Solanum* accessions are a treasured source of resistance against pathogens, including oomycete *Phytophthora infestans*, causing late blight disease. Here, *Solanum pinnatisectum*, *Solanum tuberosum*, and the somatic hybrid between these two lines were analyzed, representing resistant, susceptible, and moderately resistant genotypes, respectively. Proteome and metabolome analyses showed that the infection had the highest impact on leaves of the resistant plant and indicated, among others, an extensive remodeling of the leaf lipidome. The lipidome profiling confirmed an accumulation of glycerolipids, a depletion in the total pool of glycerophospholipids, and showed considerable differences between the lipidome composition of resistant and susceptible genotypes. The analysis of putative resistance markers pinpointed more than 100 molecules that positively correlated with resistance including phenolics and cysteamine, a compound with known antimicrobial activity. Putative resistance protein markers were targeted in an additional 12 genotypes with contrasting resistance to *P. infestans*. At least 27 proteins showed a negative correlation with the susceptibility including HSP70-2, endochitinase B, WPP domain-containing protein, and cyclase 3. In summary, these findings provide insights into molecular mechanisms of resistance against *P. infestans* and present novel targets for selective breeding.

## 1. Introduction

The late blight disease caused by *Phytophthora infestans* is the most devastating disease of potatoes (*Solanum tuberosum*) and remains a critical threat to global food security [1]. International migration, persistent oospores, fast asexual reproduction, genome plasticity, and changes caused by sexual recombination have all impacted the adaptation of *P. infestans* to plant defense mechanisms. Consequently, the resulting rapid evolution of virulence leads to reemerging late blight epidemics [2]. *P. infestans* is an obligate hemibiotrophic pathogen that attacks living tissues, and most infections during a season are initiated by rapid asexual reproduction when sporulation can occur as soon as three days after the initial leaf infection [3]. Rapid sporangia production and its long-distance dispersion by wind results in the capability of *P. infestans* to destroy unprotected crop vegetation within two weeks [2,4]. A late blight resistance resource is hidden in genotypes of wild potato species native to Mexico and the Andean highlands and developed during the ages of host and pathogen coevolution (e.g., [5]). The wild germplasm have been successfully implemented in breeding programs, including species with broad-spectrum resistance genes (e.g., *S. bulbocastanum*, *S. stoloniferum*, and *S. verrucosum*), and the number of species with reported resistance to late blight is steadily increasing [6,7,8,9,10,11,12]. However, the conventional resistance breeding process is time-consuming, giving a rapidly adapting pathogen an advantage. For instance, the introduction of *S. demissum* R genes provided only a transient resistance to the late blight, and the pathogen adapted and overcame the plant’s defense within a few growing seasons [13]. The breeding process could be considerably accelerated by effective monitoring of molecular markers associated with the resistance. However, the present-day techniques are predominantly focused on genome analyses [14,15,16,17]. These methods are cost-effective but not sufficient. Thanks to the level of ploidy and the complex nature of interactions in plant defense mechanisms, the resulting genotypic data must be correlated with the phenotypic results of the infection assays.

Fungicide application is the prevailing strategy to prevent late blight, and the chemical control increases globally, creating a selective pressure on the pathogen resulting in the emergence of fungicide-resistant isolates of *P. infestans*. Besides the significant costs spent in fungicide applications, the adverse effects of these chemicals on human health and the environment are not negligible [18]. There are new emerging strategies that could eventually replace fungicide application including the use of plant volatiles [19] and biological control by different microorganisms [20,21,22,23]. However, none of these would be as cost-effective as a new resistant genotype. Furthermore, a large-scale introduction of biological control in agriculture could represent potential biohazard, and residual pockets of the introduced microorganisms could be a threat to the natural environment. The breeding process is time-intensive and molecular markers that would facilitate the early selection of promising candidates are of paramount interest. Here, an approach employing alternative “omics” techniques was used, and molecular profiles of resistant versus susceptible genotypes were analyzed. This screening provided novel molecular markers that can be used for marker-assisted breeding and preselection of candidate-resistant genotypes.

## 2. Results

**Figure 1:**
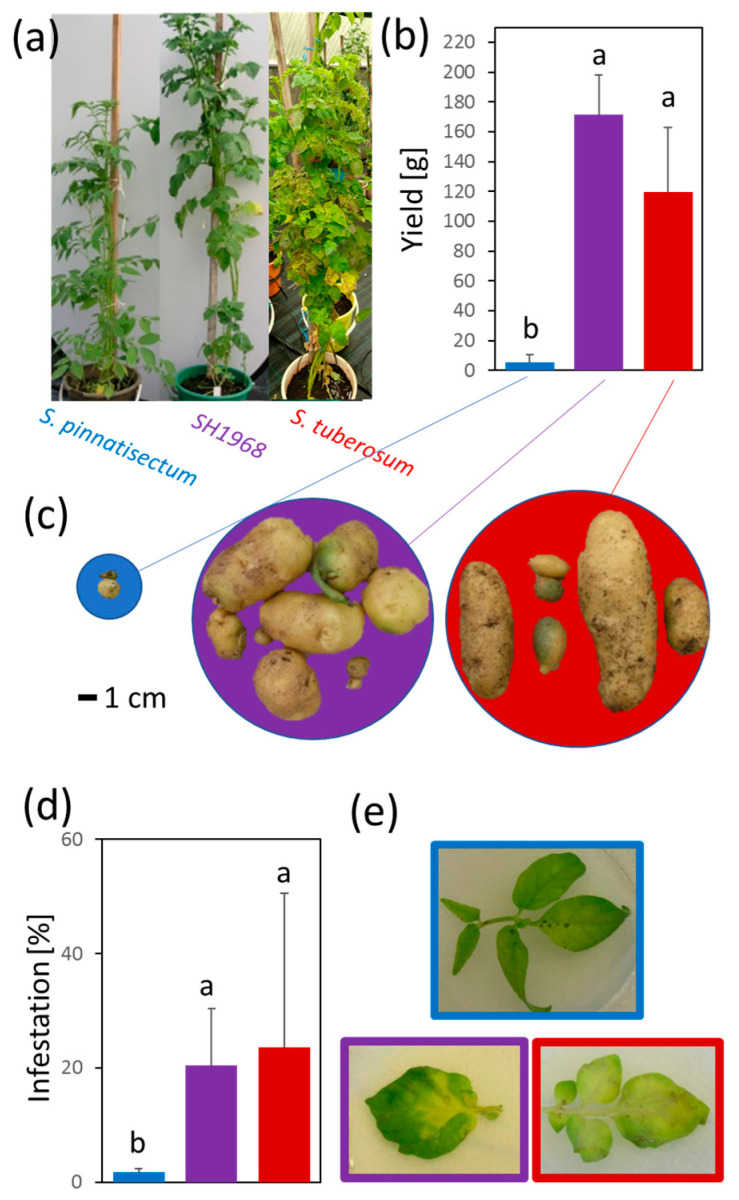
*Solanum* genotypes employed in the experiment. Representative images of 12 week old plants (**a**), comparison of yields (grams per plant) (**b**), representative images of tubers (**c**), and effects of *Phytophthora infestans* inoculation in the detached leaf experiment 96 hai (**d**,**e**). Parental genotypes *Solanum pinnatisectum* (blue), *S. tuberosum* cv. Kerkovske rohlicky (red), and somatic hybrid SH1968 (magenta). The plots represent the mean and standard deviation of four harvests (**b**) and five biological replicates (**d**). Different letters indicate significant differences (Kruskal–Wallis test, *p* < 0.05).

Three genotypes were employed in the screening experiment (Figure 1a–e), namely, *S. tuberosum* (cv. Kerkovsky rohlicek, sensitive genotype), *S. pinnatisectum* (resistant genotype), and their somatic hybrid H1968 (moderate resistance). The somatic hybrid showed similar yields to that of the commercial cultivar (Figure 1b), but its resistance to *P. infestans* was significantly promoted. Preliminary experiments showed that all plants of the sensitive genotype developed infection symptoms within two weeks after inoculation. In contrast, at least 50% of the inoculated hybrid plants were symptomless, and no symptoms were found in the resistant genotype. Next, to evaluate molecular mechanisms of resistance, the detached leaves of six-week-old plants grown in vitro were inoculated with a mixture of *P. infestans* isolates as described in Section 4. Leaf samples were collected 72 and 96 h after inoculation (hai), and the infestation level was evaluated at 96 hai by measuring the area of the lesions (Figure 1d). At this point, the difference between the sensitive genotype and the somatic hybrid was not statistically significant (Kruskal–Wallis test). 

### 2.1. The Composition of Leaf Proteome in Response to Inoculation

**Figure 2:**
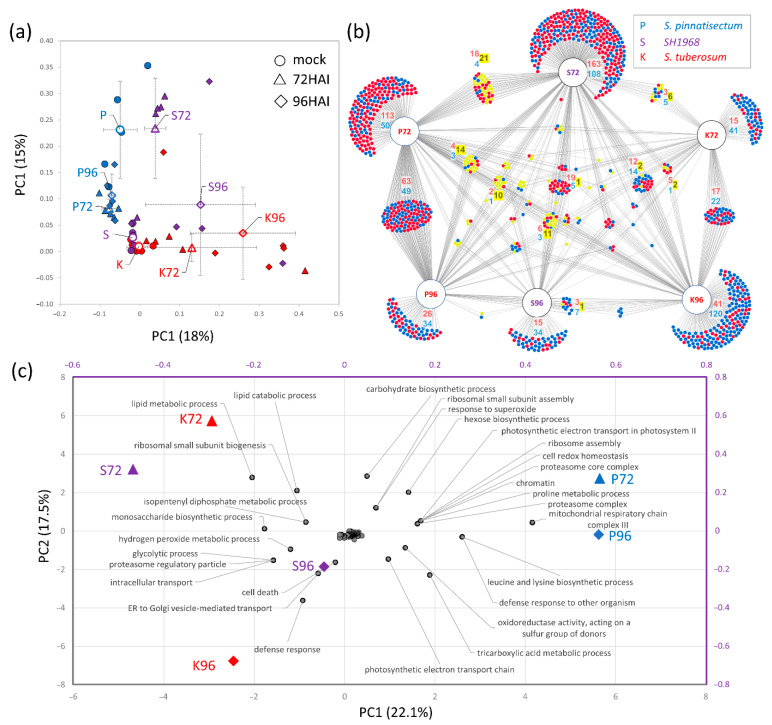
Genotype-specific response to *Phytophthora* inoculation. (**a**) Separation of proteome profiles based on relative abundances of differentially abundant proteins including the means and standard deviations. (**b**) The overlap in identified differentially abundant proteins (compared to the mock) was visualized by DiVenn [26]. Red and blue dots represent significant increases and decreases in protein abundances, respectively. Yellow dots indicate the number of proteins with a contrasting response in different genotypes and/or time points. Only proteins with at least two unique peptides were evaluated. (**c**) Comparison of enriched GO categories in the sets of differentially abundant proteins. For the sake of clarity, categories with at least five proteins were included in the final analysis, and only filtered GO terms with the most significant contribution to the separation in PC1 or PC2 are labeled. K, *S. tuberosum* cv. Kerkovsky rohlicek; P, *S. pinnatisectum*; S, hybrid SH1968. For details, see Supplementary Materials Tables S1–S3.

Five biological replicates were sampled for omics analyses. The collected samples were lyophilized and ground, and 30 mg of homogenized tissue were extracted for proteome, metabolome, and lipidome analyses. The measured spectra were searched against the *S. tuberosum* and *P. infestans* protein databases, and the final set contained 3386 and 861 protein matches, respectively. The comparison of proteome profiles showed a similarity between the mock-treated samples and the profiles of inoculated leaves of the resistant genotype. Pairwise comparisons of the inoculated and mock-treated leaves revealed a considerable number of differentially abundant proteins (*p* < 0.05, Student’s *t*-test), representing more than 40% of the data set. Interestingly, the observed effects of inoculation were mostly genotype specific (Figure 2a,b), and the estimated abundances of differentially abundant proteins represented only 6–13% of the total protein content of respective mock-treated leaves. The highest impact was found in the leaf proteome of *S. pinnatisectum* (13%), and the resistant genotype also showed the highest similarity (167 proteins) between samples collected at 72 and 96 hai (Supplementary Materials Table S1).

### 2.2. Functional Analysis of Differentially Abundant Proteins

A recent study showed that only approximately 80% of *S. pinnatisectum* genes are conserved in its relative *S. tuberosum* [24]. To at least partially compensate for the putative identification bias originating from the matching of *S. pinnatisectum* and its somatic hybrid proteome against the *S. tuberosum* database, the comparison of gene ontology (GO) enrichment analyses of all 1188 differentially abundant proteins (corresponding to 1024 Arabidopsis orthologs) was performed. The agriGO 2.0 [25] identified 625 GO terms significantly enriched in at least one sample set (Supplementary Materials Table S2), and the consecutive principal component analysis (PCA) highlighted separations of the resistant genotype and time after inoculation in the first and second principal components, respectively (Figure 2c). The analysis showed that the samples of the sensitive genotype and somatic hybrid collected at 96 hai were significantly enriched in defense response (GO: 0006952). Furthermore, the sensitive one showed twice that many differentially abundant proteins in that category (i.e., 34). The category “defense response, incompatible interaction” (GO:0009814) was represented by less than 40 proteins in all genotypes and did not contribute to the PCA separation (Figure 2c). However, most of these proteins were significantly more abundant in the sensitive genotype, their accumulation was attenuated in the hybrid plant, and six were significantly less abundant in the leaf of *S. pinnatisectum* at 72 hai. The analysis also highlighted that the Phytophthora-sensitive genotypes manifested differences in ribosome composition, cellular trafficking, hydrogen peroxide metabolism, and lipid metabolism (Figure 2c).

### 2.3. Peptide-Based Detection of P. infestans

**Figure 3:**
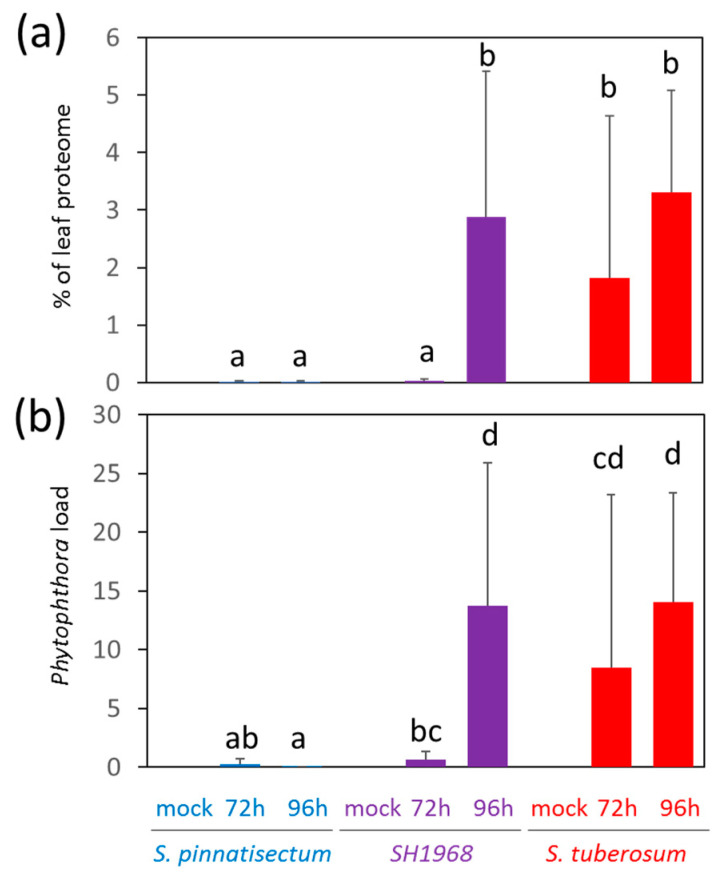
Estimated *Phytophthora* protein content in inoculated leaves (**a**) and relative *Phytophthora* load (**b**) based on the qPCR determination of *PiO8* normalized to *PGSC0003DMG400023270*. Results represent the mean and standard deviation (*n* = 5), and different letters indicate significant differences (Kruskal–Wallis, Conover test *p* < 0.05).

We have previously demonstrated that peptide-based detection of *Phytophthora* has a high sensitivity comparable to that of qPCR [27]. Similar results were found here (Figure 3a,b). In total, 706 out of 861 putative *Phytophthora* proteins were exclusively identified in inoculated samples, and these were used for *Phytophthora* content estimation. The mean *Phytophthora* content of *S. pinnatisectum* leaves was less than 0.01%, and similar values (no statistically significant difference, Kruskal–Wallis test) were found in leaves of hybrid plant SH1968 at 72 hai. In contrast, the total protein extracts of the leaves of *S. tuberosum* and SH1968 at 96 hai contained up to 7% *Phytophthora* protein. Biological variability was higher, but the differences among these three samples were not statistically significant (Kruskal–Wallis test). The comparison of identified *Phytophthora* proteins revealed a large overlap, with only 45 *Phytophthora* proteins being seemingly specific for inoculated hybrid plants. However, the available proteomic data did not provide sufficient evidence that this would be biologically relevant. 

### 2.4. Metabolomics Revealed Modulation in Both Primary Metabolism and Production of Secondary Metabolites

**Figure 4:**
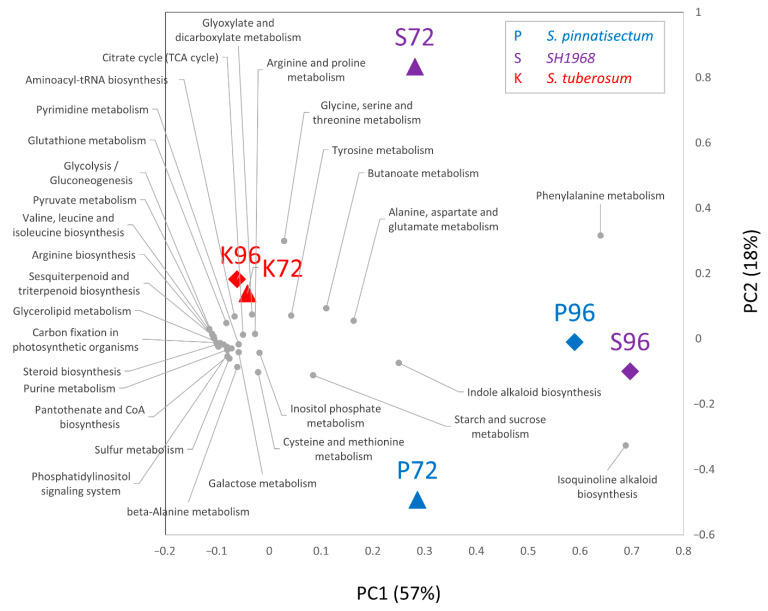
Metabolomic response to inoculation. The metabolic pathway impact based on 72 differentially abundant metabolites identified with confident identification, evaluated by MetaboAnalyst 5.0 [28], and visualized by PCA.

Polar metabolites were profiled by untargeted gas chromatography mass spectrometry (GC-MS) analysis as described in Section 4. In total, 951 compounds were resolved, and 239 passed the confident identification criteria filter (ΔRI < 5%, score > 90). The pairwise comparisons identified 281 compounds with significantly different abundances. The highest number of differentially abundant compounds (compared to the mock) was found in the resistant genotype (158). In line with the proteomics data, the response of the metabolome appeared to be genotype specific. A similar response was found in all genotypes only for 12 compounds (significantly more abundant in response to inoculation) including guanosine, adenine, uridine, ethanolamine, ribose, S-methylcysteine, gamma-aminobutyric acid, and trehalose (Table S3). The metabolic pathway analysis by MetaboAnalyst showed that the inoculation had an effect on (among others) amino acid biosynthesis (*p* < 0.01) and carbohydrate metabolism (*p* < 0.05). Interestingly, *S. pinnatisectum* and the SH1968 hybrid were enriched in the alkaloid biosynthetic pathway (*p* < 0.01), and the sensitive genotype showed an impact on TCA cycle (*p* < 0.01) (Figure 4).

### 2.5. Lipidomics Analysis Confirmed the Impact of P. infestans Inoculation on Leaf Lipidome

**Figure 5:**
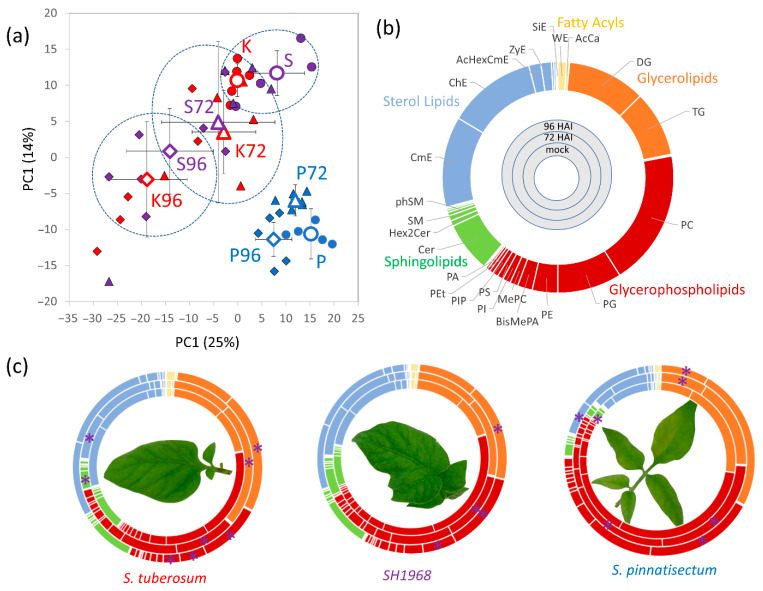
Lipidome profiling confirmed significant alterations in lipidome composition in response to *P. infestans* inoculation. (**a**) Separation of lipidome profiles by PCA, including the means and standard deviations, (**b**) estimated composition of *S. tuberosum* leaf lipidome, and (**c**) lipidome response to inoculation. Asterisks indicate statistically significant differences compared to the mock for lipid classes representing at least 2% of the estimated lipid content (Student’s *t-*test, *p* < 0.05). K, *S. tuberosum* cv. Kerkovsky rohlicek; P, *S. pinnatisectum*; S, hybrid SH1968; DG, diglyceride; TG, triglyceride; PC, phosphatidylcholine; PG, phosphatidylglycerol; PE, phosphatidylethanolamine; BisMePA, bis-methyl phosphatidic acid; MePC, methyl phosphatidylcholine; PI/PIP, phosphatidylinositol; PS, phosphatidylserine; PEt, phosphatidylethanol; PA, phosphatidic acid; Cer, ceramide; Hex2Cer, glycosphingolipids, simple Glc series; SM, sphingomyelin; phSM, sphingomyelin(phytosphingosine); CmE, campesterol ester; ChE, cholesterol ester; AcHexCmE, AcylGlcCampesterol ester; ZyE, zymosterol ester; SiE, sitosterol ester; WEs, wax esters; AcCa, acyl carnitine. For details, see Supplementary Materials, Table S4.

Proteomics analysis indicated significant differences in enzymes and proteins associated with lipid metabolism, and metabolome profiling confirmed that inoculation had an impact on metabolites of lipid metabolic pathways (e.g., ethanolamine accumulation, decrease in myo-inositol; Table S3). Thus, a shotgun lipid profiling was performed. The direct infusion analyses detected more than 650 lipid species, and fragmentation spectra provided a confident identification for at least 141 lipid compounds. The clustering of the lipidome profile by PCA showed a separation of the resistant genotype and co-clustering of mock-treated samples of somatic hybrid and sensitive genotype (Figure 5a). The most abundant compounds identified in the leaves of *S. tuberosum* were representatives of glycerophospholipids (39%), sterol lipids (29%), glycerolipids (20%), and sphingolipids (10%). In all three genotypes, glycerolipids accumulated significantly in response to inoculation and the total pool of glycerophospholipids was lower (Figure 5b,c). Interestingly, this lipidome shift was most pronounced in the sensitive genotype, with a 1.7-fold increase in glycerolipids at 72 hai. The somatic hybrid SH1968 showed a delayed response, and a statistically significant accumulation of glycerolipids was detected only at 96 hai (Student’s *t*-test, *p* < 0.05). The resistant genotype showed only a 20% increase in glycerolipids at 96 hai (Student’s *t*-test, *p* < 0.05). However, with the estimated 32.8% contribution to the lipidome composition, that was still more than in the other two genotypes. The resistant genotype and the somatic hybrid also accumulated sterol lipids at 96 hai, increasing the estimated relative content by 60 and 40%, respectively. For details, see Supplementary Materials Table S4.

### 2.6. Identification of Compounds with a Putative Role in Resistance against P. infestans 

**Figure 6:**
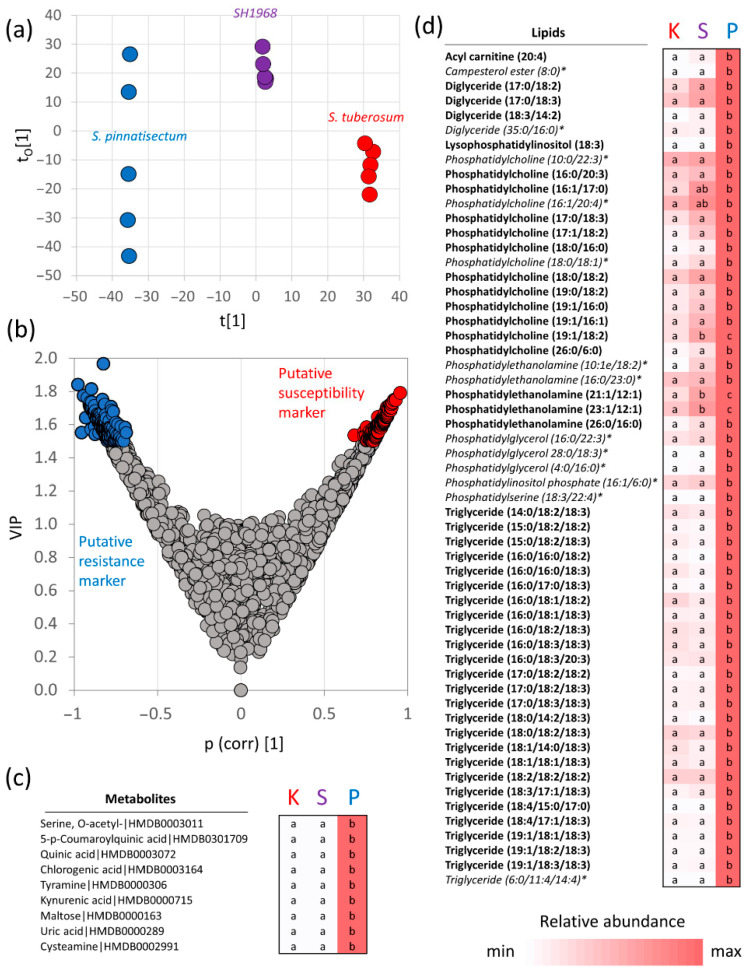
Identification of proteins, lipids, and metabolites correlating with *Phytophthora resistance*. Orthogonal partial least squares discriminant analysis (**a**) followed by VIP (variable importance in projection), (**b**) lists of identified metabolites, (**c**) and lipids (**d**) that separate the resistant genotype. Only metabolites with confident identification are shown with the corresponding HMDB metabolite identifier (https://hmdb.ca/; accessed on 10 December 2021). Heat maps represent the mean relative abundances of five biological replicates compared to *S. pinnatisectum*; letters represent the results of ANOVA and Fisher’s LSD post hoc analysis; asterisks indicate identification of lipid compounds with lower confidence. For details, see Supplementary Materials Tables S1, S3–S5. Putative protein markers are summarized in Figure 7.

**Figure 7:**
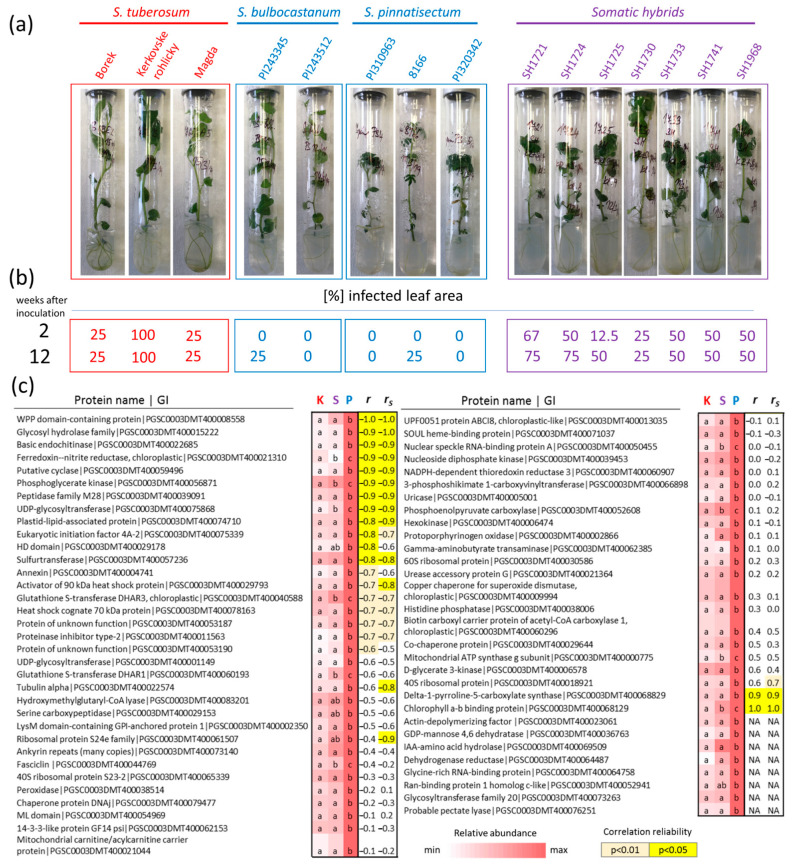
Putative protein markers of resistance to *Phytophthora* and correlation of their abundances with resistance in 15 different genotypes. Proteins identified in the analyses outlined in Figure 6a,b were quantified in an independent analysis of 15 different genotypes with contrasting resistance to *P. infestans*. (**a**) Representative images of four-week-old plants collected for proteomics analysis (before inoculation) and (**b**) the percentage of infected leaf area (developed infection symptoms) two and 12 weeks after inoculation. For details, see Section 4. (**c**) The heat map representation of the mean relative protein abundances in the leaf inoculation experiment compared to *S. pinnatisectum* and correlation coefficients (*r*, Pearson correlation coefficient; *r_s_*, Spearman’s rank correlation coefficient) representing the correlation of protein abundance with susceptibility observed in all 15 genotypes. The correlation’s reliability (*t*-test) is indicated. The protein name represents the identification determined by the orthology search against the *Arabidopsis* protein database or protein family classification determined by InterPro 87.0 [30]. GI, gene identifier according to the Potato Genome Sequence Consortium (https://solgenomics.net/; accessed on 10 December 2021). The letters represent the results of ANOVA and Fisher’s LSD post hoc analysis. NA, proteins not found in the validation experiment. For details, see Supplementary Materials Tables S1 and S5.

Next, to identify compounds with a putative role in resistance, the correlation between the observed average *Phytophthora* content at 96 hai (Figure 3a) and the molecular composition of the mock-treated leaves was analyzed via orthogonal partial least squares (OPLS) regression analysis (Figure 6a,b). That pinpointed a set of 177 molecules that positively correlated with resistance, including 64 proteins, 57 lipids, and 56 metabolic compounds (Figure 6c,d and Figure 7, Supplementary Materials Table S5). The set of proteins included enzymes of different primary and secondary metabolic pathways such as enzymes of carbohydrate metabolism, components of proteosynthesis, redox metabolism, amino acid biosynthesis, heat shock cognate 70 kDa protein (PGSC0003DMT400078163) as well as putative components of defense mechanisms, namely, basic endochitinase (PGSC0003DMT400022685) and an ortholog of glycosyltransferase 76B1 (UDP-glycosyltransferase, PGSC0003DMT400075868) that reportedly modulates salicylate and jasmonate signaling crosstalk [29]. Interestingly, 11 of these proteins were found to be *Phytophthora*-responsive in the sensitive genotype, and the abundances of ten of these increased significantly in response to inoculation (Table S1). Unfortunately, most of the highlighted metabolites were not identified, and the identification of 16 carbohydrates, an unknown steroid compound, and a phenolic compound was incomplete (Table S7).

In total, only 12 identified compounds representing nine unique metabolites were found in the set (Figure 6c). The list of highlighted lipid compounds included 26 triglycerides, indicating a putative role of lipid storage reserves in resistance (Figure 6d).

### 2.7. Targeted Analysis of Putative Protein Resistance Markers in Genotypes with Contrasting Phytophthora Resistance 

The set of 64 putative protein markers of resistance were targeted in 15 different genotypes with contrasting resistance to *P. infestans* (Figure 7a,c). In total, 56 candidates were successfully quantified, and 27 showed a negative correlation with the observed susceptibility (Figure 7c). The most prominent candidates were WPP domain-containing protein (PGSC0003DMT400008558, Pearson’s *r* = −1, *p* < 0.01), which are probably associated with the nuclear envelope and have a role in the regulation of mitotic activity and nuclear targeting; basic endochitinase (PGSC0003DMT400022685, *r* = −0.9, *p* < 0.01) which has a putative role in resistance to jasmonate-inducing pathogens [31]; putative ureidoglycolate hydrolase that belongs to the peptidase M28 family (peptidase family M28, PGSC0003DMT400039091, *r* = −0.9, *p* < 0.01); an unknown hydrolase (glycosyl hydrolase family, PGSC0003DMT400015222, *r* = −1, *p* < 0.05); a putative extracellular protein similar to cyclase-3 (putative cyclase, PGSC0003DMT400059496, *r* = −0.9, *p* < 0.01). Only two proteins showed a significant (*p* < 0.05) positive correlation with susceptibility, namely, the chloroplastic chlorophyll a–b binding protein (PGSC0003DMT400068129, *r* = 1, *p* < 0.01) and the proline metabolism enzyme delta-1-pyrroline-5-carboxylate synthase (PGSC0003DMT400068829, *r* = 0.9, *p* < 0.05). 

### 2.8. Determination of Auxin Content in Response to Inoculation

**Figure 8:**
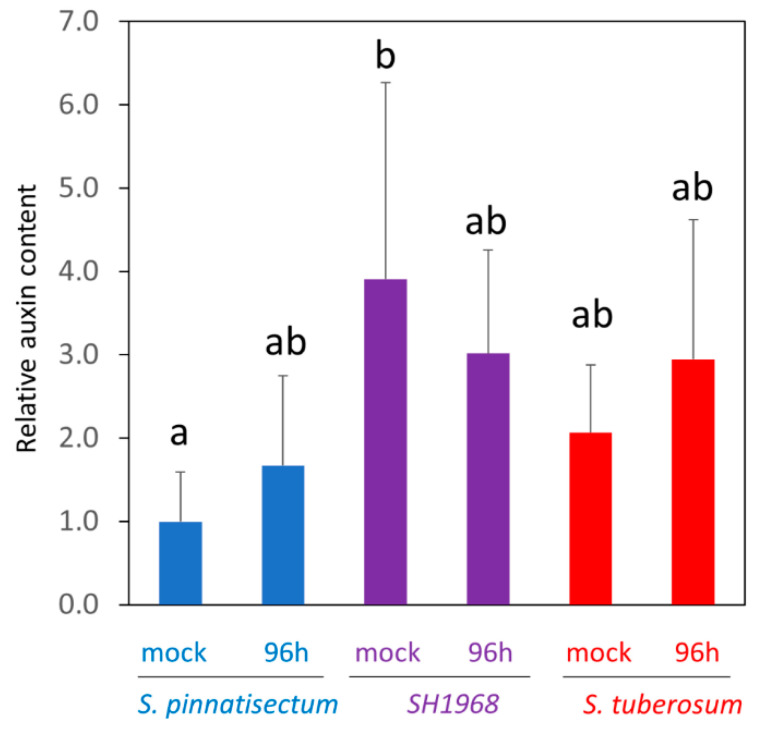
Relative auxin content in the inoculated and mock-treated leaves. The results represent the means and standard deviations (*n* = 5), and the different letters indicate significant differences (Kruskal–Wallis, *p* < 0.05). K, *S. tuberosum* cv. Kerkovsky rohlicek; P, *S. pinnatisectum*; S, hybrid SH1968.

The relative content of indole-3-acetic acid was determined in the inoculated leaves at 96 hai (Figure 8). Statistical analysis did not reveal significant differences between the mock-treated samples and the inoculated leaves. A comparison of the genotypes showed that the mean auxin content was lower in *S. pinnatisectum*, but the difference between the sensitive and resistant genotypes was not statistically significant (Kruskal–Wallis test, *p* < 0.05).

## 3. Discussion

### 3.1. The Genotype-Specific Resistance Gradient Was Attenuated but Preserved in the Detached Leaf Experiment

Preliminary experiments and disease ratings (Figure 8) consistently showed significantly promoted resistance in hybrid plants. The leaf inoculation experiment confirmed an attenuated infection progress, but resilience was not comparable to that of the intact plant. The proteome and lipidome profiles at 96 hai clustered sensitive genotype and the somatic hybrid in the same group (Figure 2a and Figure 5a), and the estimated *Phytophthora* content were not significantly different (Figure 3a,b). On the contrary, the proteome profile of the hybrid leaf at 72 hai was more similar to that of the resistant genotype (Figure 2a), and that similarity was also found in the metabolic pathway enrichment (Figure 4) and the estimated content of *Phytophthora*. However, the lower resistance is not surprising given the design of the detached leaf experiment and the fact that a low level of *P. infestans* was also detectable in inoculated leaves of the resistant genotype (Figure 3a,b).

### 3.2. The Resistant Genotype Accumulated Proline, but the Abundance of the Corresponding Biosynthetic Enzyme Did Not Correlate with the Resistance

Leaf inoculation depleted the proline pool in leaves of all genotypes. The set of proteins with a putative role in resistance to *Phytophthora* contained the delta-1-pyrroline-5-carboxylate synthase (proline biosynthetic enzyme; PGSC0003DMT400068829). It was significantly more abundant in *S. pinnatisectum* leaves, and the metabolome analysis confirmed that these leaves contained the highest amounts of proline (Supplementary Materials Table S3). Inoculation had a negative effect on the abundance of this enzyme, and both the enzyme content and proline were lower at 72 hai compared to the mock-treated controls. Interestingly, the resulting proline levels were comparable to those of the mock-treated *S. tuberosum* and were approximately four times higher than those of the inoculated leaves of the sensitive genotype. Given the well-established role of proline in stress physiology [32], it is possible that this enzyme could play a role in resistance. However, the validation experiment showed that its abundance does not seem to be a valid marker of resistance (Figure 7c).

### 3.3. Uricase Activity in S. pinnatisectum May Promote the Resistance to Phytophthora

The accumulation of nucleosides in response to inoculation was found in all genotypes. These molecules have been shown to function as signals or damage-associated molecular patterns, and an accumulation of uridine and adenosine reportedly increased susceptibility against *Botrytis cinerea* [33]. In this light, a higher abundance of uricase in *S. pinnatisectum* (PGSC0003DMT400005001) and its corresponding higher activity, evidenced in a significantly higher pool of allantoin, could provide an effective means of countering this process. In fact, compared to *S. tuberosum*, the free adenine pool in *S. pinnatisectum* was ten times lower at 96 hai (Supplementary Materials Table S3). Analysis of four-week-old plants of different genotypes confirmed that the highest abundance of uricase was found in two genotypes with the highest resistance (i.e., PI310963 and PI320342), but correlation with the resistance of all analyzed genotypes was not found (Figure 7c). 

### 3.4. The Abundance of Auxin Metabolism Enzyme Was Positively Correlated with Resistance

Protein IAA-amino acid hydrolase (PGSC0003DMT400069509) was significantly more abundant in *S. pinnatisectum* and the hybrid leaf proteome. Previous studies have found that auxin may induce resistance [34], and pretreatment with indole-3-acetic acid is reported to have attenuated the severity of the disease in potato leaves inoculated with *Phytophthora infestans* [35]. It is possible that the higher abundance of IAA–amino acid conjugate hydrolase could be correlated with resistance, but our data did not provide evidence that the total pool of indole-3-acetic acid in *S. pinnatisectum* would be significantly higher (Figure 8), and this enzyme was not detectable in the validation experiment (Figure 7c).

### 3.5. Heat Shock Protein 70 Has a Putative Role in Phytophthora Resistance

Heat shock proteins 70 (HSP70) are key components that facilitate folding, but besides their chaperon functions, HSP70s play a role in plant development and response to abiotic and biotic stress conditions including pathogen infection [36,37]. An ortholog of *Arabidopsis* HSP70-2 (Heat shock cognate 70 kDa protein, PGSC0003DMT400078163) was significantly more abundant in the resistant genotype, and its abundance was positively correlated with resistance in the set of all 15 genotypes (Figure 7c). HSP70s have previously been found to accumulate in biotic stress response (reviewed in, e.g., [38]), and orthologs of HSP70-2 were accumulated in response to *Pseudomonas syringae* in *Arabidopsis* [39], *Bemisia tabaci* in *Capsicum annum* [40], *Heterodera glycines* in *Glycine max* [41], *Synchytrium endobioticum* in *Solanum tuberosum* [42], and *Blumeria graminis* in *Triticum aestivum* [43]. The animal HSP70s play a significant role in the inflammatory and immune responses (see, e.g., [44]). It is thus tempting to speculate that a higher abundance of HSP70 in the resistant genotype(s) represents a part of the plant’s innate immunity system.

### 3.6. The Resistant Genotype Accumulated Higher Amounts of Phenolics and Cysteamine

It is well known that a decrease in phenolic compounds promotes susceptibility to *P. infestans* (e.g., [45]), and identified metabolites showing a positive correlation with resistance included quinic acid, chlorogenic acid, and 5-p-coumaroylquinic acid (Figure 6c). These compounds are known to accumulate in response to *Phytophthora* (e.g., [46,47]) and support the credibility of our OPLS model for the identification of resistance-related compounds. Tyramine (significantly more abundant in the resistant genotype) is a precursor of hydroxycinnamic acid amides, and its higher production by tyrosine decarboxylase has been reported in plants resistant to *Phytophthora* [48]. The results of proteome analysis did not provide direct evidence that the corresponding enzymes would accumulate. However, putative components of the phenylpropanoid biosynthetic pathway were more abundant in the resistant genotype including peroxidase (PGSC0003DMT400038514) and the glycosyl hydrolase family protein (PGSC0003DMT400015222) that shows a sequence similarity to *Arabidopsis* Feruloyl CoA ortho-hydroxylase. The resistant genotype also accumulated the cysteine precursor O-acetyl serine and aminothiol cysteamine that can be synthesized from cysteine through the production and hydrolysis of coenzyme A. Interestingly, cysteamine is a compound with known antimicrobial activity that reportedly sensitizes microbes to killing by reactive oxygen and nitrogen species [49]. A recent study found that treatment with cysteamine precursor, cystamine, inhibits *P. infestans* mycelial growth and sporangial germination [50]. Taken together, it seems that this compound could serve as a novel selective marker for breeding *Phytophthora-*resistant plants.

## 4. Materials and Methods

### 4.1. Plant Material, Cultivation, and Inoculation

All genotypes were obtained from the Potato Gene Bank at the Potato Research Institute Havlickuv Brod, Ltd., in the form of in vitro plants. The somatic hybrid SH1968 (2n = 6x = 72) is a product of protoplast electrofusion of the Mexican wild diploid species *S. pinnatisectum* PI320342 (pnt PI 320342, 2n = 2x = 24, 1EBN, Endosperm Balance Number) and the tetraploid *S. tuberosum* cv. Kerkovske rohlicky (tbr cv. KR, 2n = 4x = 48, 4EBN). For propagation and evaluation of yields, tubers were planted on substrate B (Raselina a.s., Sobeslav) in containers and were cultured in a net house. The plants were fertilized weekly (Hostice Slepicince), irrigated with drip irrigation, and the light regime corresponding to the Central European conditions. The growing season started with tuber planting at the beginning of May and ended with the maturity of *S. tuberosum* or artificial termination of vegetation at the end of September (pnt PI320342). The plants for leaf inoculation were grown in a growth chamber under controlled environmental conditions (22°, 16 h photoperiod, 60 µmol m^−2^ s^−1^ photosynthetic photon flux density, 60% relative humidity). The plants were grown for six weeks in modified Schenk and Hildebrandt medium, and the leaves were inoculated as previously described [27]. In summary, fully developed leaves of six-week-old plants were detached, rapidly submerged in a *P. infestans* water suspension (5 × 10^3^ spores mL^−1^, a mixture of isolates U4/16, S3/16, Z9/16, Lu2/16 kindly provided by Dr. Jana Mazáková, Czech University of Life Sciences Prague), and transferred onto a pad of damp cotton wool in a Petri dish. Leaf samples were collected 72 and 96 h after inoculation (hai) in five biological replicates and were snap-frozen in liquid nitrogen. Image analysis was performed using ImageJ 1.53e [51]. Plants for the validation experiment were cultivated in vitro on Murashige and Skoog medium supplemented with sucrose in the same growth conditions as described above and included additional 12 genotypes, namely, *S. tuberosum* cv. Magda and cv. Borek, *S. pinnatisectum* 8166 and PI310963, *S. bulbocastanum* PI243345 and PI243512, and somatic hybrids (all products of protoplast electrofusion of *S. pinnatisectum* and *S. tuberosum*) SH1721, SH1724, SH1725, SH1730, SH1733, and SH1741. The four-week-old plants were separated into two groups. The first set was used for proteomics analysis (2 × 8 plants), the second set was inoculated with *P. infestans*, and the disease rating was evaluated two weeks and twelve weeks after the inoculation. The plants were inoculated by applying sporangia suspension to the adaxial side of two fully developed leaves (one drop of 5 µL suspension per leaf with a sporangia concentration of 1.34 × 10^4^ sporangia mL^−1^).

### 4.2. Detection of P. infestans by qPCR

*P. infestans* was detected as described in [27] with minor modifications by amplifying a highly repetitive sequence PiO8 from its genome and normalization to the *S. tuberosum* gene *Ef-1α* (PGSC0003DMG400023270).

### 4.3. Proteome Analysis

The collected samples were lyophilized and ground, and 30 mg of homogenized tissue were extracted as previously described [52], while portions of samples corresponding to 5 µg of peptide were analyzed by nanoflow reverse-phase liquid chromatography mass spectrometry using a 15 cm C18 Zorbax column (Agilent, Santa Clara, CA, USA), a Dionex Ultimate 3000 RSLC nano-UPLC system, and the Orbitrap Fusion Lumos Tribrid Mass Spectrometer (Thermo Fisher, Waltham, MA, USA). Peptides were eluted with up to a 120 min, 2–72% acetonitrile gradient. Spectra were acquired using the default settings for peptide identification, employing an orbitrap analyzer with a resolution of 120,000 (MS), scan range (m/z) of 300–1700, maximum injection time of 50 ms, AGC target of 400,000, one microscan, HCD activation, 30% collision energy, resolution of 30,000 (MS2), maximum injection time of 100 ms, AGC target of 50,000, and 60 s dynamic exclusion. The measured spectra were recalibrated using a nonlinear regression model and searched against the databases of *S. tuberosum* (SolTub_3.0), *P. infestans* (strain T30-4), and common contaminants using Proteome Discoverer 2.5 (Thermo Fisher) with Sequest HT and MS Amanda 2.0 algorithms with the following parameters: enzyme—trypsin, max two missed cleavage sites; MS1 tolerance—5 ppm; MS2 tolerance—0.02 Da (MS Amanda), 0.1 Da (Sequest); modifications—carbamidomethyl (Cys) and up to three dynamic modifications including Met oxidation, Asn/Gln deamidation, and N-terminal acetylation. The chromatographic alignment and match between runs were generated by Feature Mapper (alignment: maximum RT shift 10 min, mass tolerance 10 ppm; feature linking and mapping: RT tolerance 0 min, mass tolerance 0 ppm). The quantitative differences were determined by Minora, employing precursor ion quantification followed by normalization and calculation of relative peptide/protein abundances. Only proteins with at least two unique peptides and identified in at least three biological replicates were considered for the quantitative analysis. For the validation experiment, four samples per genotype collected from ten individual plants were analyzed. The sample processing and analysis were identical, but only proteins of interest were filtered and analyzed in detail. The mass spectrometry proteomics data were deposited at the ProteomeXchange Consortium via the PRIDE [53] partner repository with the data set identifier PXD028712.

### 4.4. Metabolomic Analysis

Metabolites were extracted and fractionated with tert-butyl methyl ether/methanol mixture, derivatized, and measured using a Q Exactive GC Orbitrap GC-tandem mass spectrometer and Trace 1300 Gas chromatograph (Thermo Fisher) as described in [54,55]. Data were analyzed by Compound Discoverer 3.2 (Thermo Fisher) and searched against NIST2014, GC-Orbitrap Metabolomics library, and in-house library. Only metabolites that met stringent identification criteria (score > 90 and ΔRI < 5%) were included in the final list of identified compounds. Auxin pool was analyzed by comparing extracted chromatograms corresponding to fragments of its 2TMS derivative (*m/z* 202.1045) at the expected retention determined by its spiked deuterated analog [^2^H_5_]indol-3-acetic acid (Olchemim, Czech Republic) employing FreeStyle 1.7 (Thermo Fisher).

### 4.5. Lipidome Analysis

The lipid fraction was separated in metabolome extraction was dried by vacuum centrifugation, redissolved in 200 μL isopropanol/methanol/tert-butyl methyl ether 4/2/1 (*v/v/v*) with 20 mM ammonium formate, and analyzed by direct infusion using a Triversa Nanomate (Advion Biosciences, Ithaca, NY, USA) nanoelectrospray source. The nanospray conditions were as follows: applied voltage—1.6 kV, polarity—positive, nitrogen gas pressure—0.3 psi, sample volume taken up by tip for analysis—5 µL, and delivery time—2 min per sample. The Orbitrap Fusion Lumos Mass Spectrometer was operated in data-dependent mode (2 s cycle time), positive polarity, active dynamic exclusion, and the following parameters: sweep gas—0; ion transfer tube temperature—250 °C; MS detector type—Orbitrap; resolution—500,000; scan range (*m/z*)—150–1800; S-Lens RF Level (%)—30; AGC Target—5e4; maximum injection time (ms)—50; microscan—1; data type—profile; polarity—positive; the MS/MS analysis: Orbitrap, resolution—120,000; mass range—normal, first mass (*m/z*)—75, AGC Target—standard, maximum injection time (ms)—100, microscan—1, isolation mode—quadrupole, isolation window (*m/z*)—1.5, and activation type—HCD with stepped collision energies 25, 30, and 35%. The acquired profile spectra were analyzed using FreeStyle 1.7 and LipidSearch 4.2 (Thermo Fisher).

### 4.6. Statistical Analysis

Reported statistical tests were implemented using MetaboAnalyst 5.0 [28], SIMCA 14.1 (Sartorius), Proteome Discoverer 2.5, Compound Discoverer 3.2, STRING 11.0 [56], and MS Excel software packages. The reported quantitative differences were evaluated by the Student’s *t*-test, the background-based *t*-test, and the Kruskal–Wallis test.

## 5. Conclusions

This work provides the first integrative insight into the molecular composition of the proteome, metabolome, and lipidome in response to *Phytophthora* inoculation and presents a strategy for detecting novel molecular markers of resistance to late blight disease. It remains to be seen to what extent most of the identified compounds could be employed in the breeding program, but the available literature and the validation experiment show that at least some of the identified molecules are promising markers of resistance including metabolite cysteamine and heat shock protein 70 (heat shock cognate 70 kDa protein). 

## Data Availability

Data supporting the results are provided in the tables in the Supplementary Materials and via ProteomeXchange with the identifier PXD028712.

## Supplementary Materials

The following are available online at https://www.mdpi.com/article/10.3390/plants11010061/s1, Table S1: Results of proteomics analysis, supplementary table to Figure 2; Table S2: Gene Ontology analysis of differentially abundant proteins, supplementary table to Figure 2c; Table S3: Results of metabolomics, supplementary table to Figure 4; Table S4: Results of lipidomics, supplementary table to Figure 5; Table S5: Results of OPLS and VIP analysis, supplementary table to Figure 6 and Figure 7; Table S6: Abundances of selected proteins in 15 genotypes with contrasting resistance, supplementary table to Figure 7.
